# Analyzing and Modeling the Dynamic Electrical Characteristics of Nanocomposite Large-Range Strain Gauges

**DOI:** 10.3390/s24248192

**Published:** 2024-12-22

**Authors:** Alex M. Wonnacott, Anton E. Bowden, Ulrike H. Mitchell, David T. Fullwood

**Affiliations:** 1 Department of Mechanical Engineering, Brigham Young University, Provo, UT 84602, USA; 2 Department of Exercise Sciences, Brigham Young University, Provo, UT 84062, USA

**Keywords:** nanocomposites, viscoelasticity, high-deflection strain gauges, modeling

## Abstract

Flexible high-deflection strain gauges have been demonstrated to be cost-effective and accessible sensors for capturing human biomechanical deformations. However, the interpretation of these sensors is notably more complex compared to conventional strain gauges, particularly during dynamic motion. In addition to the non-linear viscoelastic behavior of the strain gauge material itself, the dynamic response of the sensors is even more difficult to capture due to spikes in the resistance during strain path changes. Hence, models for extracting strain from resistance measurements of the gauges most often only work well under quasi-static conditions. The present work develops a novel model that captures the complete dynamic strain–resistance relationship of the sensors, including resistance spikes, during cyclical movements. The forward model, which converts strain to resistance, comprises the following four parts to accurately capture the different aspects of the sensor response: a quasi-static linear model, a spike magnitude model, a long-term creep decay model, and a short-term decay model. The resulting sensor-specific model accurately predicted the resistance output, with an R-squared value of 0.90. Additionally, an inverse model which predicts the strain vs. time data that would result in the observed resistance data was created. The inverse model was calibrated for a particular sensor from a small amount of cyclic data during a single test. The inverse model accurately predicted key strain characteristics with a percent error as low as 0.5%. Together, the models provide new functionality for interpreting high-deflection strain sensors during dynamic strain measurement applications, including wearables sensors used for biomechanical modeling and analysis.

## 1. Introduction

Flexible high-deflection strain gauges have recently been demonstrated to be a cost-effective and accessible way to capture real-time human biomechanics in clinical settings. They have a high sensitivity factor in the range of human skin strain during typical human movement, making them ideal for biomechanical sensing [1,2]. Under static loading conditions, these nanocomposite sensors demonstrate an approximately exponential relationship between strain and electrical resistance. In this regime, they operate on the principles of quantum tunneling and percolation theory that can be predicted with computational- and/or Finite Element Analysis (FEA)-based models [3,4]. However, during dynamic strain situations, a non-monotonic and time-dependent resistance response creates difficulty for interpretation of resistance data. One of the primary challenges lies in the emergence of resistance spikes during dynamic movements. The inherent tendency of flexible sensors to drift over time due to non-recoverable creep also adds an additional layer of complexity. This study aimed to arrive at both a forward and an inverse model, relating sensor strain to resistance for cyclical-type exercises.

The forward model predicts resistance from known strain values over time, within the envelope of typical human behavior. The development of this model also gave insights into the causation behind resistance spikes, a mechanism that is unknown thus far. The inverse model, on the other hand, estimates the applied strain based on measured resistance data. During the typical usage of these sensors, the resistance data are collected, but the strain data are not directly accessible from the data; instead, characteristics of the resistance data are often correlated directly to specific motion types, for example [5]. Development of a model to determine strain values directly from resistance measurements may be vital in many instances, including clinician analysis of patient movements.

Recent studies have investigated and validated the usefulness of piezoresistive nanocomposite strain gauges in monitoring fetal movements and human hand poses [6,7], identifying sitting posture [8], measuring ligament strains [9], predicting knee angles for rehabilitation exercises [10], and phenotyping movements associated with chronic lower-back pain through biomechanical sensing [5,11]. These sensors are made from nanoparticles (usually carbon black [12], carbon nanotubes [13], or nickel nanoparticles [14]) embedded in a flexible polymer substrate (usually silicone-based elastomers [4,14,15,16] or natural rubbers [17,18]).

For the specific strain gauge sensors reported in this paper, the electrical resistance is governed by a network of nano-scale gaps between conductive particles in a silicone matrix (nickel nanostrands and nickel-coated carbon fibers embedded in Ecoflex 00-30 (Smooth-On, Macungie, PA, USA)). Electrons traverse these minute gaps via electron tunneling when gap dimensions remain within the nanometer range. Complete conduction across the entire sample ensues only when the prevalence of conducting gaps surpasses the percolation threshold [4]. When a macroscopic strain is applied, this network of gaps evolves, which, in turn, changes the electrical resistance. In static loading conditions, the strain and resistance have an inverse relationship because, under tension, the average gap size decreases due to Poisson contraction [1].

Two previously proposed models for the relationship between strain and resistance are as follows: first, there is a simple Poisson contraction (SPC) model, originally applied to these sensors by Johnson et al. [4,19,20,21,22,23,24], and second, there is a combined FEA and random resistor network model, given by Clayton et al. [3,25]. The SPC model incorporates percolation theory and Poisson contraction to predict the resistance at a given strain [14]. Proceeding with an assumption that the local strain is equal to the macroscopic applied strain, tunneling theory was used to solve the total fraction of tunneling junctions (i.e., conducting gaps) in each area of the sensor. Applying the McLachlan model, based upon percolation theory, bulk resistance can be calculated based on the fraction of tunneling junctions. Clayton et al. presented an alternative approach, where the microscopic strain and gap length were modeled with an FEA model of the sensors, and the total resistance was calculated under the assumption that the gaps between nickel nanoparticles act as a random resistor network [3]. This model proved to be more accurate than the previous model. Both models assume that the loading is static, i.e., there is no time dependence.

Baker et al. presented a quasi-static model, capturing the ‘steady state’ response of the sensors during incremental tests [26]. By fitting a spline curve to the strain–resistance relationship at the steady state points of an incremental test, Baker was able to predict the quasi-static response of the sensors. On top of this quasi-static response, he added a dynamic model that captured the spikes. He hypothesized that the spikes were related to the stress concentrations in the material, and that they could be modeled by understanding the viscoelastic behavior of the sensors. However, this research was conducted using relatively small strains, up to 15%, and at relatively low strain rates, up to 15%/s. The model performed poorly for tests with higher strains and strain rates, such as those commonly found in the human movements that are often analyzed with these sensors. Additionally, his model analyzed the response of sensors during incremental cyclic strains, which are not usually found in human movement. The new models presented here have a broader range of application, and are designed to analyze the strain–resistance relationship for all strains, strain rates, and strain accelerations found in human motion applications. In the present work, the forward and inverse models were used to analyze large-strain, high-rate sinusoidal strain gauge deformations, which are common when using wearable sensors during rehabilitation exercises or clinical assessments, for example.

## 2. Materials and Methods

This study developed a model to capture the response of a particular type of nanocomposite sensors, in the range of conditions that is likely to be found during exercises performed in clinical trials [5]. The nanocomposite sensors are composed of nickel-coated carbon fibers (NCCFs, Conductive Composites, Heber City, Utah, USA) and nickel nanostrands (NINs, Conductive Composites, Heber City, Utah, USA) mixed in the Ecoflex 00-30 silicone matrix (Dow Corning, Midland, MI, USA). The sensor recipe asks for the following material concentrations (by weight): 64% silicone, 30% NCCFs, 5% NINs, and 1% surfactant. The nanoparticles and surfactants were mixed into the silicone matrix and subsequently extruded into molds. The sensors underwent curing at 88 °C under a vacuum pressure of 650 mmHG for 135 min, followed by an additional 30 min at 192 °C. These sensors were then bonded to KT-tape (American Fork, UT, USA) with silicone, which is similar to the mounting technique used to attach the sensors to a human subject during a recent study of lower-back pain and related motion [5].

Variables of interest during human motion include changes in the skin strain and maximum strain rates. In order to study these factors and detect their influence on sensor behavior, we replicated typical human skin strain vs. time profiles using a tensile test machine. All tensile tests were strain-controlled and conducted using an Instron 3345 tensile tester (Instron, Norwood, MA, USA). The Instron was used to apply precise loads to the sensors and provide measurements of the sensor force and elongation during the loads. Polypropylene grips were applied to the sensor at either end, restricting elongation to the inner portion of the strain gauges (with an initial length, or gauge length, of 20 mm). A pretension of approximately 0.1 N (~3% strain) was applied to the sensors prior to the start of each tensile test to avoid the initial slack. The sensors’ electrical DC resistance during these tests was measured using an NI 9215 data-acquisition device (National Instruments, Austin, TX, USA). The sensors were prestrained for ten cycles between the initial strain and 30% strain to minimize the potential impact of the Mullins effect [26]. The effects of temperature and humidity have been shown to cause variations in the effectiveness of the sensors, so all tests were performed at room temperature and under similar humidity conditions (~40% relative humidity) [27].

**Figure 1:**
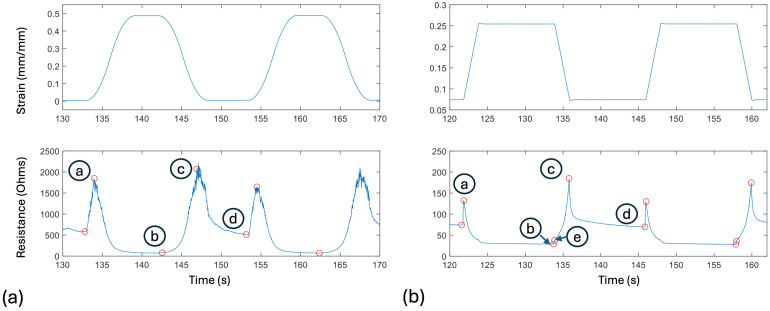
(**a**) Strain profile for sinusoidal-type tests (**top**) used to mimic human movement during dynamic movements, with associated sensor resistance response (**bottom**). (**b**) Square-wave tests used for comparison with the smooth sinusoidal-type. Red circles indicate peaks and troughs of the resistance profile. Several of these are labeled (a–e) for later discussion. Point e indicates the top of a small ‘instantaneous’ rise in resistance during a strain decrease that is present for the square wave, but not the sinusoidal, tests.

To capture the key features of human motion, motion capture data were obtained from sensors placed on the back of a human subject while the subject engaged in 14 different movements, designed to reflect the skin’s strain response during a range of human movements. These movements included both traditional uniplanar activities, like spinal flexion and extension, as well as functional movements, like sit-to-stand [5]. From these motion capture data, it was found that individual sensors experience up to 50% strain during a movement, with a maximum strain rate of 60%/s, and an average cycle time of 7 s. These tests also revealed that the skin strain curve was roughly sinusoidal for the specified exercises, but with pauses at the top and bottom of each cycle. These characteristics were recreated on the Instron tensile tester, as shown in Figure 1.

### 2.1. Forward Model Development

Due to the complex relationship between resistance and strain for these sensors, straightforward linear models, and even sophisticated viscoelastic models, prove inadequate in comprehensively capturing the interrelationship. Unlike typical viscoelastic stress relaxation behavior, which would be typified by a rapid stress increase, followed by a slow relaxation after a positive change in strain, the resistance spikes are positive during both increasing or decreasing strain changes; hence, the positive resistance spike during a drop in strain cannot be correctly captured within a simple viscoelastic model.

To capture all aspects of the resistance response during a cyclic test, four different sub-models were developed. First, a linear regression model was used to represent the quasi-static relationship between strain and resistance. Second, a phenomenological model was implemented to predict the spike magnitude, based upon a combination of different variables, including strain rate and strain acceleration. Third, a decay model was created, dealing with the resistance changes due to the short-term, recoverable viscoelastic behavior of the sensors after a given spike. Lastly, a creep decay regression model was formulated to deal with the resistance changes due to the long-term, unrecoverable creep behavior of the sensors. These models were then combined to create a complete forward model to extract the resistance relating to a given strain path.

The complexity of the model creates difficulty in directly inverting the forward model. Hence, an inverse problem approach is applied to extract key strain variables, such as the maximum strain rate and strain acceleration, from key resistance variables, such as the spike magnitude and decay rate. These key strain variables are then used to produce a prospective sinusoidal strain curve that can be input into the forward model to test for accuracy in an iterative manner.

#### 2.1.1. Quasi-Static Linear Regression Model

**Figure 2:**
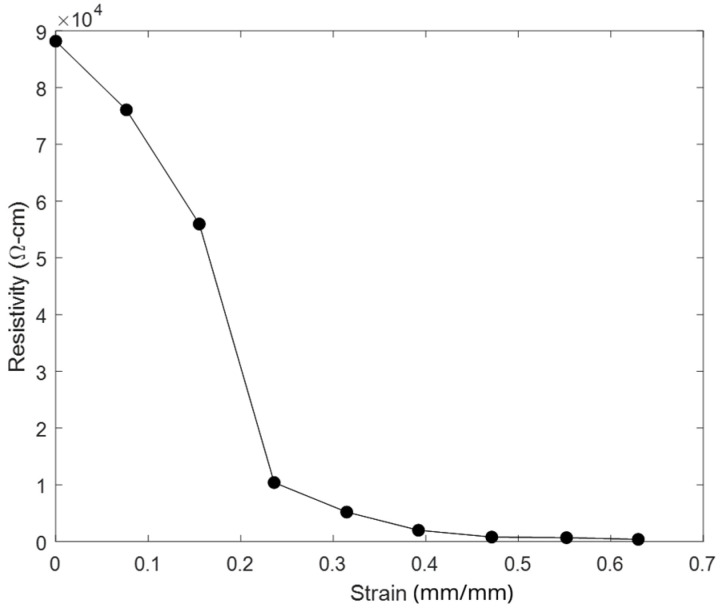
Quasi-static resistivity–strain experimental data of a typical sensor. This curve follows a shape similar to a logistic curve across the entire strain range. Adapted from data presented in [3].

Different methods have been used to model the static and quasi-static relationship between the strain and resistance of these nanocomposite sensors. Johnson et al. and Clayton et al. showed that the resistivity–strain curve exhibits characteristics similar to a logistic function across a wide range of strains, as shown in Figure 2 [3,4]. Baker et al. showed that, at small strains and during the quasi-static plateaus of incremental cyclic-type exercises, the resistance–strain relationship exhibits the behavior of an exponential curve [26]. By fitting quasi-static points with a spline tool, they were able to create an accurate model of the resistance vs. strain under those conditions. They also observed that under certain small ranges of strains, the strain–resistance relationship is approximately linear. Although the overall static strain–resistance curve is generally non-linear, for simplicity, this paper assumes that, under cyclic strains, the quasi-static strain–resistance relationship is approximately linear. A quasi-static condition is assumed to approximately occur at points b and d in Figure 1, the points at which the decay has had the longest time to asymptote; these are also the points directly before the next resistance peaks. These ‘steady state’ points are used for the calibration of the desired linear model, as follows: R=aε+b, where R is sensor resistance and ε is its strain. MATLAB (version R2023a)’s “fit” tool is used to calculate the constants *a* and *b*. Because of the variability in the microstructure and the related response of the sensors, the model must be calibrated for each individual sensor (potentially from quality control data for each sensor).

#### 2.1.2. Spike Magnitude Model

Four factors were used to characterize the resistance spikes that occur upon a change in strain path. The factors were calculated from selected points on the resistance curves, as shown in Figure 1a. The points *R*_a_ (the resistance at point a) and *R*_c_ represent the peak of the two resistance spikes during a given cycle, of which the first relates to an increase in strain and the second relates to a decrease. Points *R*_b_ and *R*_d_ capture the positions of minimum resistance following the spikes. Point *R*_e_ in Figure 1b relates to a change in slope of the resistance spike during a rapid decrease in strain that is present on the ‘square wave’ tests, but not on the smoother sinusoidal-type tests. The absence of this slope change during realistic strain paths led to the abandonment of the sharp strain changes during square-wave tests, and the adoption of the sinusoidal tests as being more representative of actual human testing.

The equations used to obtain the spike characteristic factors, *F_i_*, in terms of the resistance value associated with each point, are as follows: *F_1_* = *R*_c_/*R*_d_; *F_2_* = *R*_a_/*R*_b_; *F_3_* = (*R*_c_ − *R*_b_)/(*R*_d_ − *R*_b_); *F_4_* = *R*_a_/*R*_d_. *F_1_* and *F_3_* relate to the spikes during increasing strains, while *F_2_* and *F_4_* relate to decreasing strain. Using these four factors, correlations were investigated between spike magnitude and initial strain ($\varepsilon_{0}$), change in strain (Δε), hold time ($t_{h}$, the time period for which the sensor is held at a constant strain), strain rate ($\dot{\varepsilon }$), and strain acceleration ($\ddot{\varepsilon }\;$). To examine correlations, strain-controlled tests were undertaken while varying target variables and holding others constant (where possible). No strong positive or negative correlations were found between the spike magnitude and $\varepsilon_{0}$ or Δε, so these variables were not used in the final model prediction. Correlations with maximum strain rate, ${\dot{\varepsilon }}_{max}$, and maximum strain acceleration, ${\ddot{\varepsilon }}_{max}$, were then explored, while the decay time was held constant. It was found that both ${\dot{\varepsilon }}_{max}$ and ${\ddot{\varepsilon }}_{max}$ have positive correlations with the spike magnitudes captured by the factors defined above. These positive correlations were then quantified by fitting them to a linear curve of the form $F_{i}=k_{1}{\dot{\varepsilon }}_{max}+k_{2},\;or\;F_{i}=k_{1}{\ddot{\varepsilon }}_{max}+k_{2}$, where $F_{i}$ is a spike magnitude factor, and $k_{i}$ are constants. These fits were performed with MATLAB’s “fit” tool, depending upon which parameter has the best correlation with the given factor. With these constants, the spike magnitude factors can be predicted, given the maximum strain and the maximum acceleration of a sensor.

**Figure 3:**
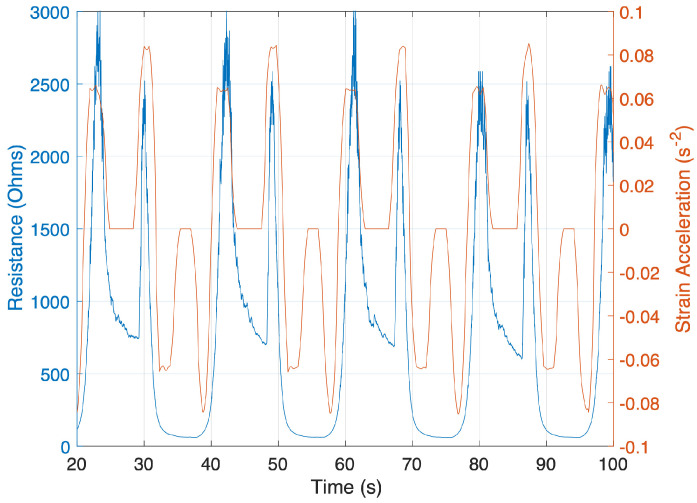
Resistance response (blue) to the applied strain acceleration (orange). This overlay demonstrates the precise alignment of strain acceleration peaks and resistance peaks.

This spike magnitude model predicts the relative height of the resistance spike based on the ‘steady state’ portion of the strain data. However, the location and timing of the resistance spikes are not captured. To predict the timing of the resistance spikes, strain acceleration is used. As can be seen in Figure 3, the timing of the resistance peaks corresponds directly to the timing of the positive strain acceleration peaks. With this correlation, the previously calculated spike magnitudes can be added to the quasi-static linear model at the appropriate times.

#### 2.1.3. Short-Term Decay Model

**Figure 4:**
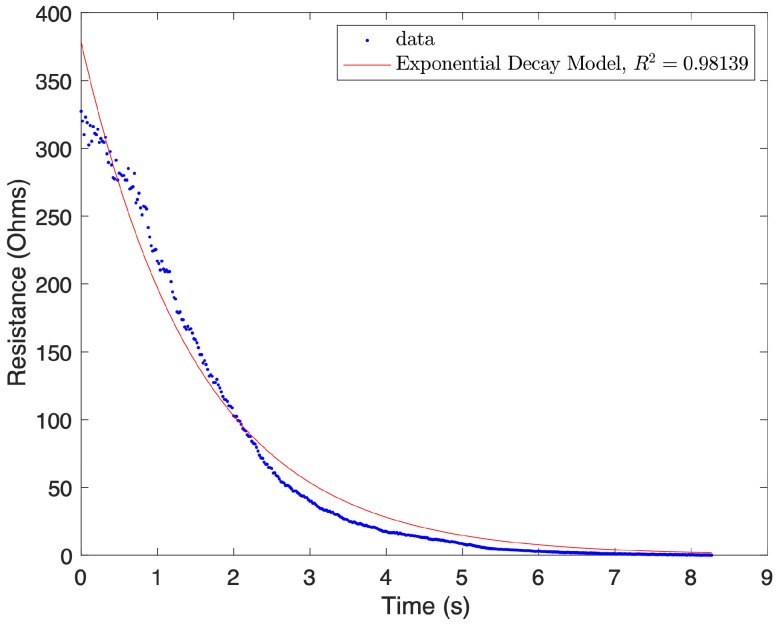
Data from the fourth cycle of a selected sensor from points a–b in Figure 1. A first order exponential decay equation has been fitted to the data.

Previous studies have found that, after a resistance spike, the decay of the resistance is related to the viscoelastic stress relaxation of the sensors, which can be modeled with a simple first order exponential decay model [26,27]. Hence, a first order exponential decay model was used to model the short-term, recoverable viscoelastic behavior of the sensors, with the form of $R=R_{0}{\mathrm{e}}^{k_{3}t}$. The initial resistance spike magnitude, $R_{0}$, is obtained from the previously calculated spike magnitude model, so the rate constant, $k_{3}$, was the variable of interest. To find these rate constants, each cycle was separated into the following two sections: the increasing strain spike and decay (letters a, b in Figure 1), and the decreasing strain spike and decay (letters c, d in Figure 1). Using MATLAB’s “fit” tool, the exponential model was fitted to these data, and the rate constant was extracted. These rate constants were then compared to their respective spike magnitude factors, and were found to have strong negative linear correlations with them. The relationship between the rate factor and the spike magnitude factor were then fitted with a first-degree polynomial curve fit, again using MATLAB’s “fit” tool. These linear curve fits between the spike magnitude and rate constant were used to predict the rate constant of the sensors, given the spike magnitude calculated with the spike magnitude model. Figure 4 demonstrates the accuracy of the model for a specific case.

#### 2.1.4. Long-Term Creep Decay Model

Viscoelastic creep has been shown to cause exponential decay in the resistance of the sensors over time [26]. This is somewhat mitigated by the preconditioning cycles that move the sensors beyond primary creep, but it still has a small effect throughout the testing. Because of this, a first order exponential decay function was used to model the overall decay of the spike magnitudes with respect to time, with the form of $R=R_{a,1}{\mathrm{e}}^{kt}$, with *R* being the spike magnitude, $R_{a,1}$ being the magnitude of the initial spike, and k being the rate constant. Because the creep is a variable for each sensor, a calibration was taken with an initial test, which can then be used for all models with that same sensor. As previously stated, two decay models were calibrated, one for increasing and one for decreasing strain spikes. To create the calibrated creep decay model, the exponential model was fitted to the resistance spikes (points a and c from Figure 1a) and their corresponding times, using MATLAB’s “fit” function. To apply the model, the initial magnitude $R_{a,1}$ is taken from the first spike of the previously calculated spike magnitude model, and the rate constants $k_{7},\;k_{8}$ from the calibrated model are used to calculate the rest of the resistance spikes. The results of this model are then combined with the results of the spike magnitude model to calculate the final predicted spike magnitudes.

#### 2.1.5. Combined Model

The linear, spike magnitude, and decay models were combined to form one comprehensive model that aims to represent all aspects of the resistance data. As will be shown in a later example, the spike magnitude factors, $F_{i}$, are determined from a knowledge of the following key exercise characteristics: $\varepsilon_{0},\;$Δε, ${\dot{\varepsilon }}_{max}$, ${\ddot{\varepsilon }}_{max}$, and hold time, $t_{h}$. These factors enable the calculation of $R_{a,1},R_{b,1},R_{c,1},R_{d,1}$, from the relations above. They then feed into the mathematical model that represents the complete model, which is as follows:

(1) $$R\left(t\right)=\left\{\begin{array}{c} k_{1,n}\varepsilon +k_{2,n},\;\;\;\;\;\;\;\;\;\;\;\;\;\;\;\;\;\;\;\;\;\;\;\;\;\;\;\;\;\;\;\;\;\;\;\;\;\;\;\;\;\;\;\;\;\;\;\;\;t_{d,n-1}<t<t_{a,n}\\ \begin{array}{c} R_{a,n}=\frac{\left({\dot{\varepsilon }}_{m}-k_{5}\right)R_{d,1}/k_{6}+R_{a,1}{\mathrm{e}}^{k_{7}t}}{2},\;\;\;\;\;\;\;\;\;\;\;\;\;\;\;t=t_{a,n}\\ R_{a,n}{\mathrm{e}}^{k_{3}t},\;\;\;\;\;\;\;\;\;\;\;\;\;\;\;\;\;\;\;\;\;\;\;\;\;\;\;\;\;\;\;\;\;\;\;\;\;\;\;\;\;\;\;\;\;\;\;\;\;t_{a,n}<t<t_{b,n}\\ \begin{array}{c} k_{9,n}\varepsilon +k_{10,n},\;\;\;\;\;\;\;\;\;\;\;\;\;\;\;\;\;\;\;\;\;\;\;\;\;\;\;\;\;\;\;\;\;\;\;\;\;\;\;\;\;\;\;\;\;\;\;\;t_{b,n}<t<t_{c,n}\\ R_{c,n}=\frac{\left({\dot{\varepsilon }}_{m}-k_{11}\right)R_{d,1}/k_{12}+R_{c,1}{\mathrm{e}}^{k_{8}t}}{2},\;\;\;\;\;\;\;\;\;\;\;\;\;\;\;t=t_{c,n}\\ R_{c,n}{\mathrm{e}}^{k_{4}t},\;\;\;\;\;\;\;\;\;\;\;\;\;\;\;\;\;\;\;\;\;\;\;\;\;\;\;\;\;\;\;\;\;{\;\;\;\;\;\;\;\;\;\;\;\;\;\;t}_{c,n}<t<t_{d,n} \end{array} \end{array} \end{array}\right.$$

with times a, b, c, and d referring to points on the curve found in Figure 1a, and *n* referring to the cycle number. Linear models (the first and fourth equations) are adjusted at each cycle to connect points d and a, and points b and c. The spike magnitude and creep decay models are combined to find the height of the resistance spike at times a and c, and the short-term decay model is used to find the resistance between times a and b, and times c and d. In terms of validation, the complete calibrated model was used to capture the resistance of given tests with three different tests with differing maximum strain rates. An R^2^ value was calculated for each test and used to determine the goodness of fit of the model.

### 2.2. Inverse Model Development

Due to the intricate nature of our comprehensive forward model, directly obtaining an inverse for predicting the strain from measured resistance poses a challenge. However, an inverse problem approach is applied to help predict key strain metrics from resistance data. Key resistance metrics of spike magnitude and decay rate are taken from the given resistance data and used to evaluate their causation. A two-step method is utilized for extracting strain data from a given resistance.

#### 2.2.1. Step 1: Correlation-Based Key Variable Extraction

Four key strain variables are required for defining the assumed sinusoidal-square-wave strain curve, as follows: Δε, ${\dot{\varepsilon }}_{max}$, ${\ddot{\varepsilon }}_{max}$, and $t_{h}$. Resistance variables (the four factors, F_i_, defined above) can be extracted from the resistance data that have correlations to these key strain variables. These four spike magnitude factors are extracted, and a first order exponential decay model is used to extract the hold time, $t_{h}$, of the sensors. Using previously identified correlations between the spike magnitude and maximum strain rate and acceleration, the maximum strain rate and strain acceleration can be predicted. Additionally, using the calibrated linear curve, the change in strain and cycle time can be extracted as well. These four factors, once extracted, hold potential significance for clinicians. This approach allows for a practical interpretation of resistance data to be applied to clinically relevant strain metrics, providing a valuable avenue for real-world applications.

#### 2.2.2. Step 2: Inverse Problem Strain Curve Reconstruction

The general shape of the applied strain curve is that of a square-wave, with the peaks and troughs connected by sinusoidal portions. The values of Δε and $t_{h}$ defined the peaks and troughs of the curve, while the values of ${\dot{\varepsilon }}_{max}$ and ${\ddot{\varepsilon }}_{max}$ are employed to determine the sine function parameters for connecting the peaks and troughs. These values are extracted at step 1 and provide the initial estimate for the strain curve. This strain curve is then used as the input into our strain–resistance model (Equation (1)), generating a predicted resistance curve. The predicted resistance curve is then compared to the given resistance, and the key metrics of the provisional strain curve are iteratively adjusted until the output resistance of the forward model closely aligns with the actual experimental resistance. This iterative process refines the strain curve and the key strain metrics, ultimately producing a strain–resistance relationship that closely mirrors the experimental data.

The model was validated using experimental data, where strain and resistance values were simultaneously measured. Assuming that the strain data were unknown, the resistance data were used to extract key strain features and then to predict a full strain curve. The key strain curve features were iteratively optimized after running the predicted curve through the forward resistance prediction model multiple times. The key factors were evaluated based on the percent error. The predicted strain curve was evaluated based on the R^2^ value of the curve vs. the actual strain data.

## 3. Results

### 3.1. Forward Model

This model is calibrated on the strain and resistance data from a sensor undergoing cyclical sine wave tests with a maximum strain rate of 50%/s and a maximum strain acceleration of 10%/s^2^. These parameters are within the bounds of human movement found earlier in the study. The linear and creep decay models are fit to these calibration data, and these calibrated fits, along with the two general models of spike magnitude and viscoelastic decay, are used to complete the forward model (Equation (1)). We report on the process of generating this model below, with an analysis of the accuracy of each sub-model.

#### 3.1.1. Calibrated Quasi-Static Linear Model

Calibrating the linear regression model on 30 sensors (10 cycles per sensor) resulted in an R^2^ value of 0.99. This linear model is the base to which the other three models are added.

#### 3.1.2. Spike Magnitude Model

**Figure 5:**
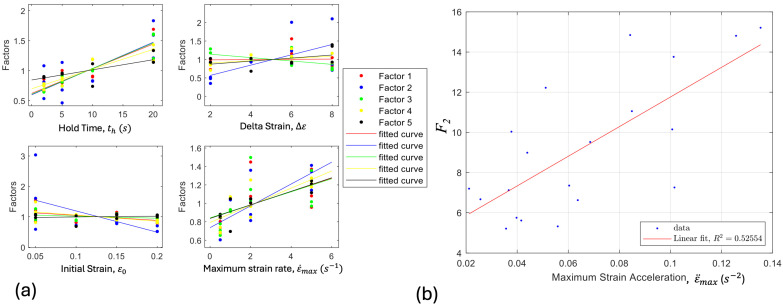
(**a**) Correlations between $t_{h}$, Δε, $\varepsilon_{0}$, and ${\dot{\varepsilon }}_{max}$ and spike magnitude factors, *Fi*. (**b**) Sample correlation between ${\ddot{\varepsilon }}_{max}$ and *F*_2_. R-squared values for all correlations are shown in Table 1.

Cyclical tests were performed on the 30 different sensors with hold times, $t_{h}$, ranging from 2–20 s, Δε of 10–50%, $\varepsilon_{0}$ of 0–20%, ${\dot{\varepsilon }}_{max}$ of 2.5–50%/s, and ${\ddot{\varepsilon }}_{max}$ of 2–20%/s^2^. As shown in Figure 5a, Δε and $\varepsilon_{0}$ exhibit no significant correlation with spike magnitude factors, while ${\dot{\varepsilon }}_{max}$, $t_{h}$, and ${\ddot{\varepsilon }}_{max}$ have positive linear correlations with spike magnitude factors. Corresponding R-squared values can be found in Table 1. An example of a linear fit between F_2_ and ${\ddot{\varepsilon }}_{max}$ is shown in Figure 5b. Based upon the definitions of these spike factors and the measured values of ${\dot{\varepsilon }}_{max}$ and ${\ddot{\varepsilon }}_{max}$, the spike magnitudes are calculated and added to the quasi-static values from the previous model.

#### 3.1.3. Short-Term Decay Model

**Figure 6:**
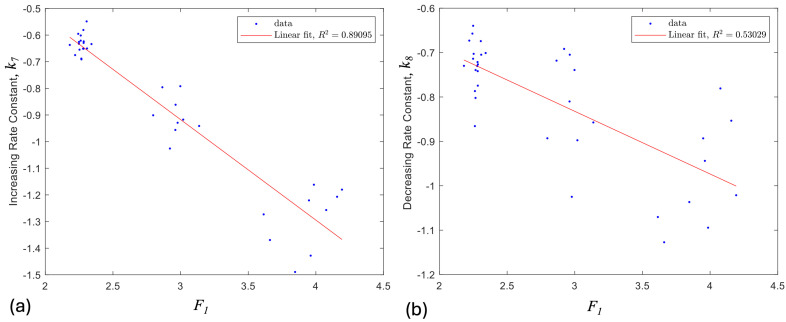
Example of linear correlation between the spike magnitude factors and the rate constants. (**a**) Correlation between increasing strain rate constant, $k_{3}$, and *F_1_* for the 30 sensors tested. (**b**) Correlation between decreasing strain rate constant, $k_{4}$, and *F_1_*. R^2^ values are shown in Table 1.

An exponential decay equation was fit to the top and bottom spike decays for each cycle of the exercises described in the previous section (30 sensors and multiple parameter values), as illustrated in Figure 4. The rate constants from these equations were extracted and compared to the spike magnitude factors of their corresponding spikes. The rate constants were found to have strong linear correlations with the spike magnitude factors, as shown in Figure 6. The increasing spikes and decay were found to have stronger correlations than the decreasing spikes and decay, as demonstrated by the R^2^ values in Table 1. These correlations are used in this model to predict the rate constants for the decay following the resistance spikes. The rate constants are found by inputting the predicted spike magnitude factors found in the previous models into the general model, correlating spike magnitude factors to rate constants. Using the equation $R=R_{0}{\mathrm{e}}^{kt}$, with the rate constant *k*, and using the spike magnitude predictions from the spike magnitude and creep decay models to provide $R_{0}$, the decay after the resistance spikes is predicted. This decay is then added to the previous models.

#### 3.1.4. Long-Term Creep Decay Model

Based upon the same test data used for the other model components, two exponential calibration curves were fitted, one for the gradual decrease in height of the increasing strain spikes, and one for the decreasing strain spikes. Calibrating the increasing spike and decreasing spike creep decay models resulted in exponential curve fits with R^2^ values of 0.56 and 0.34, respectively. Using these calibrated equations and the predicted spike of the first cycle obtained from the spike magnitude model, the magnitude of the rest of the spikes in the series are calculated.

#### 3.1.5. Forward Model Validation

**Figure 7:**
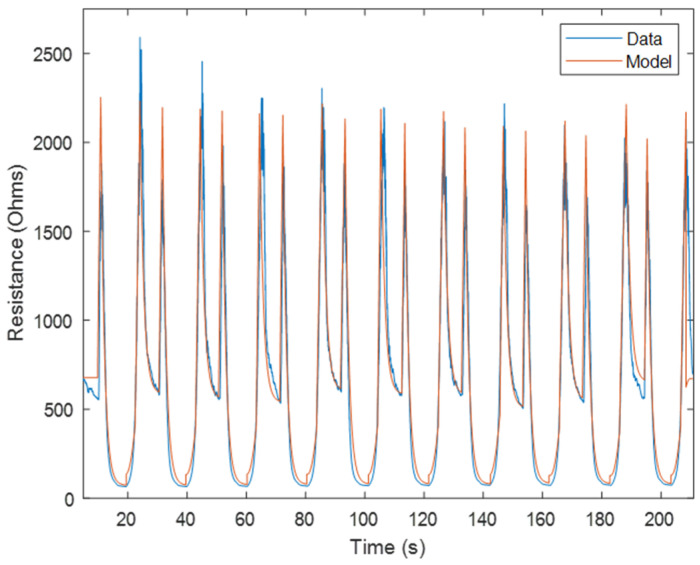
Complete model of resistance predicted from strain, with an R-squared value of 0.897. This model combines linear, spike magnitude, long-term creep relaxation, and short-term viscoelastic decay relaxation models into one complete resistance prediction model.

The model was validated by performing multiple cyclical sinusoidal tests with the same sensor that was used for calibration. The validation tests were conducted with maximum strain rates of 15%/s, 30%/s, and 50%/s. With the strain data from the validation tests and the calibrated linear model, an initial quasi-static linear model was formed. The plateaus, or steady state points, of this linear model are then selected and used as the inputs to the generalized spike magnitude model to calculate the spike magnitudes. The strain acceleration data from the validation tests are used to determine the location of the spikes with respect to time. With the spikes aligned, the spike magnitudes from the first cycle are used as inputs into the calibrated creep decay model. The magnitudes of the creep decay spikes are then averaged with the magnitudes from the general spike magnitude model, and are added to the calibrated linear model. Using the spike magnitude factors obtained from the linear and spike magnitude models, along with the general short-term decay model, the rate constants for the short-term decay are determined and used to add in the decay after the resistance spikes, resulting in the complete model seen in Figure 7. The models fitted for the validation tests were calculated to have an average R^2^ value of 0.88, which is better than the R^2^ of 0.80 that the model by Baker et al. had at similar strain rates [26]. Table 2 shows the R^2^ values for three different tests that this model was fitted to, all of which are above 0.85, indicating that they fit the model very well. The points of maximum error in this model are the increasing strain peaks, which contained an error of up to 17%.

### 3.2. Inverse Model

Solving a complex inverse model is required for predicting important strain measurements from resistance data. Due to the intricate nature of the complete forward model, directly obtaining an inverse for predicting strain from resistance poses a challenge. Obtaining a complete strain curve from any set of resistance data from this model is impossible. However, because the approximate shape of the strain curve is known to be sinusoidal and cyclic, a prediction of the strain curve can be produced by focusing on particular aspects of the strain–resistance relationship and building a model from these aspects.

#### 3.2.1. Inverse Model Results

The inverse model was calibrated with the same strain and resistance data as the forward model. For the inverse model, the goal was to produce a sinusoidal-square-wave curve from the resistance data that resemble the actual sine curve. To do this, the resistance data were taken, and points were selected at the peaks and troughs, as shown in Figure 1. These peaks and troughs were then used to calculate the four spike magnitude factors for the data. An exponential decay equation was also fitted to each of the cycles, and the decay rate factors were extracted from those equations. The decay rate factors were found to have correlations with the spike magnitude factors, and those correlations were used to extract spike magnitude factors from the decay rate factors. These two sets of spike magnitude factors, one from the resistance data and one from the decay rate correlations, are then averaged for the best fit. The spike magnitude factors were previously found to have correlations with the strain rate and strain acceleration. The averaged spike magnitude factors are input into these correlations, and predictions for the maximum strain rate and maximum strain accelerations are extracted. The troughs of the resistance data are used as inputs to the calibrated quasi-static linear model to predict the change in strain, and the distance between two troughs is used to calculate the cycle time. Thus, four key strain curve features are predicted, as follows: change in strain, maximum strain rate, maximum strain acceleration, and cycle time. With these four factors, a provisional sinusoidal strain curve is created. This curve is then input into the complete forward model, and the outputted resistance is compared to the original resistance that we started with. After comparing the two resistance curves, the key factors of resistance were adjusted, and the strain curve was again input into the forward model, where the resistance curves can be compared. This process was repeated multiple times, until the forward model produced a resistance curve with an R^2^ greater than 0.85.

#### 3.2.2. Inverse Model Validation

The validation tests that were used to assess the performance of the forward model were also used to validate the performance of the inverse model. The same calibration was also used. Strain values are predicted by evaluating the relationship between key resistance factors and key strain factors. The calibrated linear model, along with the quasi-static resistance troughs, were used to predict the change in strain. The spike magnitude factors, along with the rate decay factors, were used to predict the maximum strain rate and maximum strain accelerations. The cycle time was also predicted using the troughs of the resistance data.

**Figure 8:**
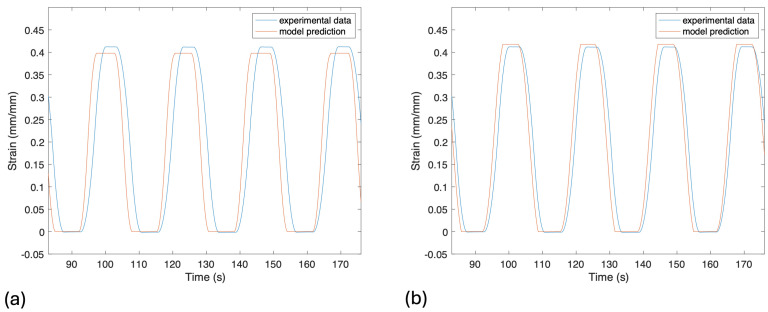
(**a**) Initial and final predictions of strain curve. The strain curve predictions are created from the following predicted key strain factors: Δε, ${\dot{\varepsilon }}_{max}$, ${\ddot{\varepsilon }}_{max}$, and $t_{h}$. The initial strain curve prediction has an R^2^ value of 0.696. (**b**) After iteration through the forward model, the final predicted strain curve has an R^2^ value of 0.905. The iterations through the forward model improved the accuracy of the model.

The inverse model was validated in the following two ways: first, by analyzing the percent error of the key strain factors before and after iterations of running the strain curve through the forward model, and second, by calculating the R^2^ value of the predicted vs. experimental strain curves. Initially, the delta strain was accurate, but the maximum strain rate and the maximum strain acceleration predictions were not accurate, as can be seen in Table 3. After iteration, the error percentage greatly improved from the initial guess. The delta strain was able to decrease to below a 1% error, while the maximum strain rate and maximum strain acceleration were both below a 5% error. The initial R^2^ for the strain prediction was 0.696, while the R^2^ for the prediction after the iterations was 0.905, as shown in Figure 8. The accuracy of the inverse model was greatly improved by iteratively adjusting the key strain factors to match the resistance output of the forward model.

## 4. Discussion

This study explores the relationship between the strain input and resistance output of high-deflection nanocomposite strain gauges. The results align with prior research, indicating that viscoelastic sensors display highly non-linear outputs [15,26]. This study also advances the existing knowledge by correlating the relationship between strain and resistance in both quasi-static and high strain rate dynamic conditions, particularly during sinusoidal cyclical strains.

### 4.1. Model Review

Previous attempts to model the strain–resistance relationship of nanocomposite sensors used physics-based principles, such as percolation theory and quantum tunneling, to model the static resistance response of the sensors [4,19,20,21,22,23,24]. Others used internal geometry and FEA to predict the resistance of static sensors [3,25]. The method used in this research produced a purely mathematical model, similar to the one devised by Baker et al., that fully captures the resistance response of the sensors under both quasi-static and dynamic conditions [26]. This mathematical model incorporated methods of modeling creep and viscoelastic elements found in the mechanical modeling of viscoelastic polymers [28]. The resulting model was validated to determine its ability to correctly interpret the resistance of sensors from strain measurements. The model was able to predict the resistance output of a sensor under sinusoidal cyclical loading in various situations with high accuracy (average R^2^ of 0.88), as shown in Table 2. With only one calibration test, the model was able to accurately predict the resistance response during tests with differing maximum strain rates and accelerations.

The forward model was composed of the following four parts: the quasi-static linear model, the spike magnitude model, the short-term decay model, and the long-term decay model. The quasi-static linear model and the long-term creep decay model were both determined to be sensor-specific, and they were calibrated for each sensor individually. The short-term decay model and the spike magnitude model are general models that apply to all sensors, inasmuch as the motion of the sensors is within the range of human movement. They were both modeled by fitting the exponential decay and linear equations, respectively, to the resistance and strain data of multiple sensors. These general models are only applicable to sensors of the same recipe. Adjusting the recipe adjusts the sensitivity factor of the sensors, which changes the height of the spikes. The short-term decay model only applies to sensors that have been bonded to KT-tape, while the spike magnitude model applies to all sensors of the same recipe. Because the KT-tape affects the viscoelastic recovery of the sensors, modeling unbonded raw sensors or sensors with other coatings would require the fitting of a new short-term decay model.

The inverse model, used to convert resistance data to strain data, was created through the use of an inverse problem, extracting correlations between key resistance factors and key strain factors. This model is key for real-world applications of the nanocomposite sensors, because, in clinical settings, the resistance of the nanocomposite sensors is much easier to collect than strain data; however, strain data are the most useful to clinicians. This inverse model is made using the same linear calibration as the forward model. Peaks and troughs extracted from the resistance data are used to calculate the spike magnitude and decay rate factors. Correlations between these factors are leveraged to predict strain curve features like maximum strain rate and acceleration. The calibrated quasi-static linear model predicts strain change and cycle time. These key strain factors are used to generate a sinusoidal strain curve. Iterative adjustments to resistance factors are made until the forward model achieves an R^2^ greater than 0.85 in generating the resistance curve. The resulting predicted strain curve had an R^2^ of 0.91, and the key strain factors had an average percent error of 2.0.

As can be seen in Figure 8, the most significant difference between experimental and predicted strain lies in a small temporal shift between the two. This may relate to the ambiguity in determining position b in Figure 1, where the point of change in strain directionality is somewhat difficult to identify exactly on the resistance graph. Better algorithms for identifying such points on the resistance graph (possibly exploiting characteristics of the first and second derivatives of the resistance) might reduce this error.

This inverse model is capable of predicting the maximum strain rate of a symmetrical sinusoidal strain curve. However, human movement is not always symmetrical, and, in fact, one of the key interests for clinicians when evaluating patients is the difference between the maximum strain rate and acceleration for the increasing and decreasing strain portions of the cycle. These differences are of great importance to clinicians, as they allow the clinician to make inferences about the potential range of motion restrictions and/or causes of pain. While this model does not take into account the asymmetric nature of human movement, it contains the necessary data to identify and create separate correlations between the increasing and decreasing strains. By separating the spike magnitude factors into increasing and decreasing factors, separate correlations can be used to create an asymmetrical strain curve approximation.

The main intent of the model is to be able to effectively reproduce key strain characteristics of the sensors from resistance data that have been collected during in-clinic, rehabilitation-type exercises. Because the skin strain during these exercises is cyclic in nature, this model is effective only when applied to sinusoidal cyclic motion. To interpret the resistance response during general human motion, other factors would have to be considered.

### 4.2. Resistance Spike Interpretation

While various models have been created to simulate the resistance response of these nanocomposite strain gauges, the mechanism behind the spikes in resistance during positive strain accelerations is previously unstudied [3,4,26]. It is assumed that the resistance of the sensors is determined by the geometry of the nickel nanoparticles throughout the silicone matrix [29,30]. When the sensors are static, effective conductive paths are formed along the paths of least-resistance through the sensor [31,32,33]. When the geometry of the sensors changes during dynamic movements, the conductive paths are broken or changed, causing the resistance to temporarily increase until new conductive paths are formed. The following two different types of resistance spikes were detected during testing: acceleration spikes and instantaneous spikes. Acceleration spikes are so-named because they line up with the peaks in strain acceleration; they are found during all dynamic motions of the sensor and occur over an appreciable time span, relating to the applied acceleration. Instantaneous spikes occur very rapidly (~50 ms) and were detected during square-wave tests at the point of ‘instantaneous’ strain change; for these tests, both types of spikes can be observed; the instantaneous spikes were not found during sinusoidal-square-wave tests, where the acceleration is closer to the values seen during human motion. Instantaneous and acceleration spikes can be found in Figure 1, with instantaneous spikes only on the square-waves and acceleration spikes on both plots. Hypotheses are presented for both types of resistance spikes.

One hypothesis for the causation of acceleration spikes is that stress concentrations between fibers create abnormalities during the contraction and expansion of the bulk material. At the stress concentrations between particles, at microscopic level, Poisson contraction of the silicone matrix between nanoparticles is occurring at different rates for different directions. If the fiber orientation and tensile direction affect the rate of Poisson contraction, then there would be a sudden change in the resistance of the effective conductive paths before the formation of new effective conductive paths. Another explanation for the differing rates of Poisson contraction is the presence of voids in the material. A CT scan of the sensors confirmed the presence of voids amidst the silicone matrix, despite the vacuum-sealed manufacturing process. With the presence of voids, the volume of the material does not remain completely constant, allowing for extension and contraction in different planes to happen at different rates. This hypothesis is supported by the fact that when the strain rate increases, the spike magnitude also increases. The difference in the Poisson contraction in different directions is magnified at larger strain rates. Additionally, the decay time affecting the spike magnitude also supports this hypothesis. Poisson contraction is driven by the stresses between gaps, and the decay time increases the recovery of the stress within these gaps. With a larger decay time, the stress would be more uniform throughout the sensor, meaning that there will be a larger change in the Poisson contraction during the next cycle.

The instantaneous spikes can be explained by a disconnection between the nickel fibers and the silicone matrix. The disconnection and introduction of voids in between the silicon and the nickel would cause a sharp increase in resistivity and cause the effective conductive paths to break at the point of tension. The rate of reconnection, if high, explains the absence of instantaneous spikes during lower strain accelerations (i.e., sinusoidal curves). This also lines up well with the observation that the spike magnitude increases with higher strain accelerations, because the larger changes in strain rate would lead to more dislocations occurring at the junctions between nanofiber and silicone matrix.

## 5. Conclusions

This study presents a robust and accurate model for predicting resistance vs. time output for viscoelastic nanocomposite strain gauges, based on strain vs. time measurements. Consisting of four parts, the model captures the quasi-static relationship, dynamic resistance spikes, viscoelastic decay, and creep decay portions of the strain–resistance relationship. Given the relevant calibration data for a given sensor during a cyclical test, the combined model can predict the resistance output of that sensor at any strain, strain rate, and strain acceleration within the realm of human movement with an R^2^ greater than 0.85. The proposed approach to the inverse problem, when applied to cyclical strain data of a sinusoidal nature, offers a practical means to derive a complete strain curve from a given resistance curve, unlocking the interpretability of sensor data. Based upon an accurate forward model that predicts resistance data from strain data, the inverse model approach iterates over key strain features, such as strain amplitude, strain rate, and strain acceleration, to arrive at the strain cycle that would produce the measured resistance data. The inverse model prediction had an R^2^ of 0.91, and the predicted key strain factors had an average percent error of 2.0. The capacity of the model for predicting the sensor strain from experimental resistance data is particularly valuable for understanding intricate aspects of human movement in biomechanical applications. Furthermore, observations of the nature of resistance spikes presented here contribute theoretical insights into the mechanisms behind this curious behavior. It is believed that some resistance spikes are caused by a difference in Poisson contraction, caused by stress concentrations at the nano-junctures of the particles or by voids in the material, while the instantaneous spikes are caused by a dislocation between the nanoparticles and the silicone matrix.

To apply this model to non-cyclical and non-uniform data, it is important to remember that the correlations that were found between the different strain variables and resistance spike magnitudes apply across all sensor movements. If any patterns or trends can be found in the movement, the sensors can be calibrated, and mathematical equations can be developed to model these particular trends or patterns. While the model presented in this research is calibrated to fit cyclical, sinusoidal data, the framework for developing new models that can be calibrated to fit any number of strain curves has also been demonstrated. Overall, the findings demonstrate the practical utility and versatility of the proposed model for analyzing sensor data in the context of human movement, thereby offering valuable tools for clinical applications and biomechanical research.

## Figures and Tables

**Table 1:** R-squared values for correlations between spike magnitude factors and different variables.

Variable	*F_1_* R^2^	*F_2_* R^2^	*F_3_* R^2^	*F_4_* R^2^
$\mathrm{Hold}\;\mathrm{Time},\;t_{h}$	0.777	0.593	0.883	0.838
Delta Strain, Δε	0.001	0.344	0.311	0.188
$\mathrm{Initial}\;\mathrm{Strain},\;\varepsilon_{0}$	0.180	0.329	0.101	0.387
$\mathrm{Strain}\;\mathrm{Rate},\;{\dot{\varepsilon }}_{max}$	0.627	0.602	0.540	0.620
$\mathrm{Strain}\;\mathrm{Acceleration},\;{\ddot{\varepsilon }}_{max}$	0.515	0.526	0.427	0.472
$\mathrm{Increasing}\;\mathrm{Rate}\;\mathrm{Const},\;k_{3}$	0.530	0.585	0.518	0.549
$\mathrm{Decreasing}\;\mathrm{Rate}\;\mathrm{Const},\;k_{4}$	0.891	0.903	0.868	0.881

**Table 2:** R^2^ values for the fit of the complete strain–resistance model. All models were created from a calibration test with a maximum strain rate of 50%/s.

	R^2^
15%/s maximum strain rate test	0.853
30%/s maximum strain rate test	0.897
50%/s maximum strain rate test	0.892

**Table 3:** Error of key strain factors before and after iterations through the complete forward strain–resistance model.

	Initial % Error	% Error After Iterations
Delta Strain	3.84%	0.51%
Maximum Strain Rate	22.7%	2.25%
Maximum Strain Acceleration	54.7%	3.25%

## Data Availability

Processed data are contained within the article. Examples of raw data and some plotting functions are shared at this location: Fullwood, David (2024), “High elongation strain sensor data used in Sensors manuscript”, Mendeley Data, V2, doi: 10.17632/9jrjswxtbf.2.
